# The Comparison between Circadian Oscillators in Mouse Liver and Pituitary Gland Reveals Different Integration of Feeding and Light Schedules

**DOI:** 10.1371/journal.pone.0015316

**Published:** 2010-12-15

**Authors:** Isabelle M. Bur, Sonia Zouaoui, Pierre Fontanaud, Nathalie Coutry, François Molino, Agnès O. Martin, Patrice Mollard, Xavier Bonnefont

**Affiliations:** 1 CNRS, UMR 5203, Institut de Génomique Fonctionnelle, Montpellier, France; 2 INSERM, U661, Montpellier, France; 3 Université Montpellier, Montpellier, France; Pennsylvania State University, United States of America

## Abstract

The mammalian circadian system is composed of multiple peripheral clocks that are synchronized by a central pacemaker in the suprachiasmatic nuclei of the hypothalamus. This system keeps track of the external world rhythms through entrainment by various time cues, such as the light-dark cycle and the feeding schedule. Alterations of photoperiod and meal time modulate the phase coupling between central and peripheral oscillators. In this study, we used real-time quantitative PCR to assess circadian clock gene expression in the liver and pituitary gland from mice raised under various photoperiods, or under a temporal restricted feeding protocol. Our results revealed unexpected differences between both organs. Whereas the liver oscillator always tracked meal time, the pituitary circadian clockwork showed an intermediate response, in between entrainment by the light regimen and the feeding-fasting rhythm. The same composite response was also observed in the pituitary gland from adrenalectomized mice under daytime restricted feeding, suggesting that circulating glucocorticoids do not inhibit full entrainment of the pituitary clockwork by meal time. Altogether our results reveal further aspects in the complexity of phase entrainment in the circadian system, and suggest that the pituitary may host oscillators able to integrate multiple time cues.

## Introduction

Internal circadian clocks govern daily variations in gene expression, physiology and behavior. In mammals, the main circadian pacemaker resides in the suprachiasmatic nuclei (SCN) of the anterior hypothalamus. A complex interplay between cell-autonomous rhythmic properties of SCN neurons and their network organization ensures the robustness of this central clock [1]. At the molecular level, the SCN display circadian rhythms in transcriptional activity [2], and mutated alleles of so-called clock genes such as Period (Per1 and Per2), Cryptochrome (Cry1 and Cry2), Clock and Bmal1 alter the circadian outputs from the SCN [3], [4], and circadian locomotor behavior [5]. Thus, a consistent ensemble of molecular and cellular oscillators within the SCN drives overt rhythms at the level of the organism.

Interestingly, clock genes also tick outside the SCN, both in the brain and peripheral organs [6], [7]. Recently, cell-type specific targeting of altered clock gene alleles revealed the physiological relevance of peripheral oscillators in the retina [8], heart [9], liver [10], [11] and pancreas [12]. It is worth noting that only few of all circadian transcripts in the liver remain rhythmically expressed in absence of a functional clock in hepatocytes [10]. Hence, the tissue-specific circadian program in gene expression, representing up to 10% of the total gene transcripts in a given organ [13], mostly relies on local oscillators rather than systemic cues driven by the SCN.

Therefore, a key question is to understand how these multiple clocks get together within the organism, and how they adjust to daily changes of the external world. The ambient light-dark cycle and the feeding schedule are important time cues (zeitgebers) able to entrain circadian oscillators [14]. Light is undoubtedly the most potent zeitgeber, and resets the SCN pacemaker through direct retino-hypothalamic inputs [1], [14]. Conversely most peripheral clocks, but not the SCN, are entrained by meal time [15], [16], [17]. A dichotomy thus appears, dividing the circadian system in light-tracking and food-entrained clocks, respectively. How these various time cues are integrated to promote the cohesion of body clocks is still puzzling. Importantly, the SCN are necessary to synchronize peripheral oscillators [18], and thus stand at the top of the hierarchical circadian system. But the synchronizing mechanisms along the clockwork web remain unclear, although both nervous and humoral factors have been proposed to mediate SCN timing to the rest of the body, including action through the autonomous nervous system [19], [20], and circulating glucocorticoids [21], [22].

The pituitary gland is a tempting candidate to convey at least part of the SCN control to peripheral clocks. Indeed, the pulsatile secretion of pituitary hormonal products in the main bloodstream tightly depends on specific hypothalamic neurons that receive direct or indirect inputs from the SCN [23]. Interestingly, hypophysectomy induced alterations of daily profiles in body temperature or feeding behavior in rats subjected to time-restricted feeding [24]. Moreover, the pituitary gland also exhibits rhythmic expression of circadian clock genes and proteins [25], [26], [27] that are independent from the SCN and persist in explants cultured ex vivo [18], [28], [29]. However, the functional significance and the regulating factors of the pituitary clockwork have not been documented to date. In the present study, our goal was to investigate the regulation of circadian clock gene expression in the pituitary gland, and make a comparison with the liver as a peripheral oscillator of reference, to decipher whether this endocrine interface between the brain and other organs behaves like the majority of other peripheral circadian clocks. Our results reveal a complex response of the pituitary clock genes to changes of photoperiod or meal schedule. This suggests that the gland clockwork integrates both light- and food-associated cues, and thus may act as a relay between the SCN central pacemaker and peripheral circadian oscillators.

## Results

### Photoperiod differentially alters clock gene expression in the liver and pituitary gland

**Figure 1:**
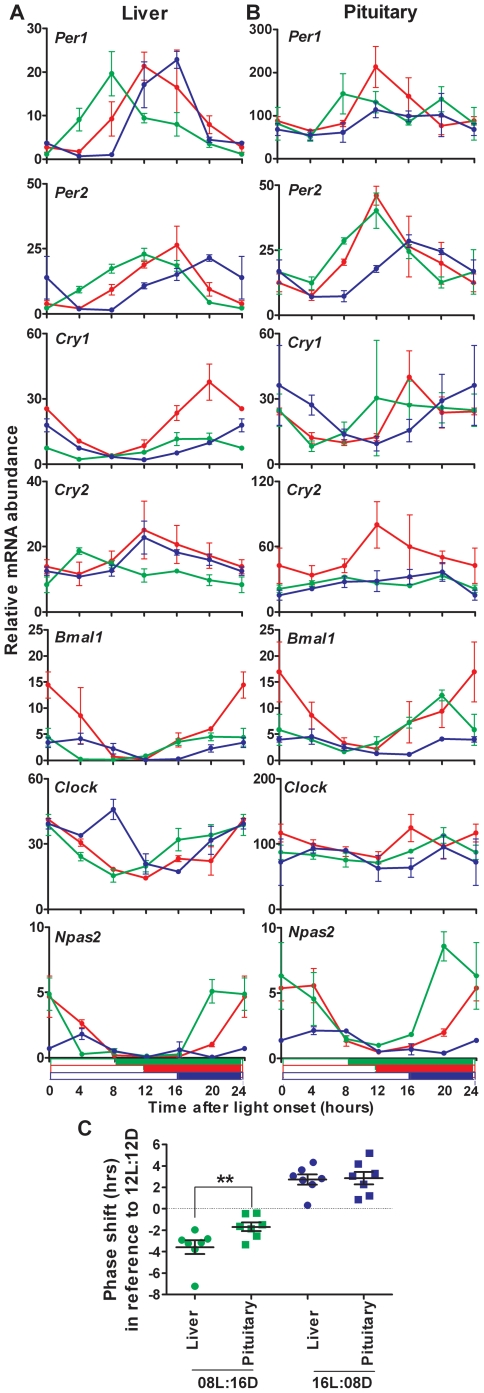
Expression profiles of circadian clock genes in liver and pituitary under three different photoperiods. Accumulation of clock gene transcripts was assessed by real-time quantitative PCR at six time points in the liver (**A**) and pituitary gland (**B**) of male mice raised under 08L:16D (green), 12L:12D (red) or 16L:08D (blue) light schedules, respectively. Values obtained at lights on have been plotted twice (t = 0 and t = 24) for better visualization. Data are plotted as mean ± standard error of the mean, n = 3 or 4 at each time point. The open and solid bars below graphs indicate the duration of the light and dark phases for each photoperiod, respectively. (**C**), The estimated phase- shift, as compared to the 12L:12D light cycle was calculated for each gene profile under short (green dots) or long (blue dots) photoperiod. The average shift was significantly different between liver and pituitary under 08L:16D conditions (**, p<0.01).

As reported previously [25], when mice were raised in a symmetric 12-hour light: 12-hour dark cycle (12L:12D), we observed daily variations in expression of all the genes tested that were very similar in the pituitary gland and the liver (Figure 1A and 1B, profiles in red). Note that whereas rhythmic activity of the circadian clockwork in the pars tuberalis region depends on melatonin signaling [30], circadian clock gene expression is cyclic in the rest of the pituitary gland of C57/Black6 mice, normally devoid of melatonin [31]. The estimated phase of each gene pattern was calculated by cosinor analysis, and we found no significant difference between the liver and pituitary (p = 0.23, Wilcoxon matched-pairs signed rank test), suggesting that the oscillators of both organs are synchronized. Only few differences were noticeable in the mean relative levels of expression of some clock genes that appeared higher in the pituitary gland, as indicated by the midline estimating statistic of rhythm (MESOR) values of Clock (25.05±1.12 vs. 100.69±4.03), Per1 (17.35±1.17 vs. 111.52±8.66) and Cry2 (8.15±1.07 vs. 51.49±4.39). Moreover, the elevated accumulation of Clock transcripts in the pituitary goes along with an apparent dampening of their daily fluctuation, as compared to the liver (Figure 1B).

Under long (16L:08D) and short (08L:16D) photoperiod, several changes were noted in the liver and pituitary gland, as compared to the 12L:12D condition. These changes mostly consisted in phase shifting, and reduced amplitude for some clock gene oscillations, without alteration in the overall waveforms at our time resolution. We thus decided to use cosinor analysis to evaluate the relative phase of transcript accumulation between each light condition. Globally, under the 16L:08D condition, a delay in the peak of expression could be observed for each clock gene in both organs (Figure 1A and 1B). The calculated phase delays between the 12L:12D and 16L:08D regimen ranged from 0.34 hrs (Cry2) to 4.31 hrs (Per2) in the liver, and from 0.85 hrs (Bmal1) to 5.18 hrs (Cry1) in the pituitary. In average the liver and pituitary oscillators were delayed by 2.72±0.47 hrs and 2.86±0.59 hrs, respectively (Figure 1C, p>0.05). Thus, the circadian oscillator in the liver and the pituitary gland experienced a phase delay under long photoperiod that is equivalent in both organs.

Interestingly, although we also observed phase-shifts, the response to short photoperiod differed between pituitary and liver oscillators. Indeed, under the 08L:16D schedule, the circadian clock genes reached their maximum of expression earlier after light onset than under the 12L:12D cycle (Figure 1A and 1B). However, the estimated phase advance ranged from 1.96 hrs (Cry1) to 7.24 hrs (Cry2) in the liver, and from 0.40 hrs (Clock) to 3.37 hrs (Bmal1) in the pituitary gland. The mean overall advance was 3.59±1.70 hrs in the liver, and only 1.69±1.06 hrs in the pituitary (Figure 1C, p<0.01, Wilcoxon matched-pairs signed rank test, and repeated measures ANOVA followed by Tukey's Multiple Comparison Test). Thus, the liver and pituitary clocks that oscillated in phase under the 12L:12D and 16L:08D schedules became desynchronized under a 08L:16D cycle, with an average lead of 2 hours for the liver clock.

**Figure 2:**
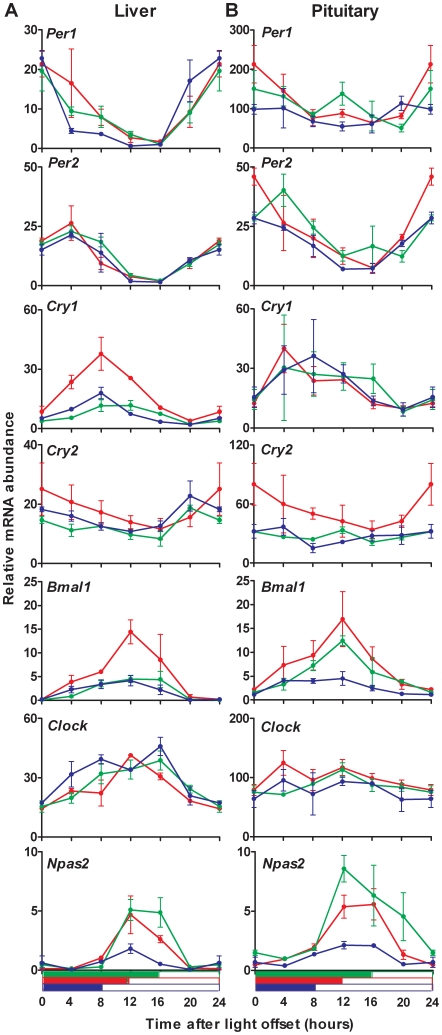
The circadian oscillator in liver, but not in pituitary, was phase-locked to light offset. Data presented in Figure 1 were re-plotted, with the time origin defined as light offset. Note the better alignment of the various gene expression profiles in the liver (**A**) than the pituitary (**B**). Values obtained at lights off have been plotted twice (t = 0 and t = 24) for better visualization. The symbol and color legend is as described in Figure 1.

The gradual phase shifting observed for the liver clock when mice were raised under all three light-dark schedules roughly corresponded to the increase of day length. This prompted us to verify whether the gene expression profiles obtained under 08L:16D and 16L:08D regimen could be superposed with that from the 12L:12D condition, simply by sliding them by 4 hours toward the right or left hand, respectively. Indeed, the re-plotting of our data with the time origin defined as light offset instead of light onset, revealed a clear alignment of gene patterns in the liver (Figure 2A). To estimate the goodness of the superposition between the expression patterns, we calculated the deviation between the average profile of each gene under the 12L:12D condition and the experimental data for the corresponding gene under either 08L:16D or 16L:08D schedules. For both photoperiods, the sum of residual errors was decreased for all genes when data were plotted with the new timescale, as compared to the initial plotting, and the overall alignment was significantly improved (p<0.01, Wilcoxon matched-pairs signed rank test). Hence, the liver clock was tracking cues associated with light offset (i.e. the onset of locomotor and feeding activity for nocturnal mice), which is in agreement with food intake being the main zeitgeber for the liver clock.

On the contrary, the re-plotting of data from pituitary glands on a new timescale based on light offset did not improve the alignment of circadian clock genes under either photoperiod (Figure 2B). Globally, the sum of residual errors was not significantly reduced (p>0.05 for both 08L:16D and 16L:08D, Wilcoxon matched-pairs signed rank test). The marked alteration under long and short photoperiods in the amplitude of most transcripts rhythm of accumulation, especially the almost loss of overt rhythmicity for Per1 and Cry2 in the pituitary and not in the liver (Figure 2A and 2B), might partly account for this result. The regulation of circadian clock gene expression by photoperiod therefore differed between the pituitary gland and the liver. The pituitary clock was not set by a unique time cue associated either to light onset or offset, which suggested that food intake (or other signals associated to light offset) might not be a major zeitgeber for the pituitary, as it actually is for the liver and other peripheral clocks.

### Daytime restricted feeding dampens the apparent rhythm of the pituitary oscillators

To test this hypothesis that meal time would be a weak entraining cue for the pituitary circadian oscillators, we submitted mice to temporal feeding restriction under a 12L:12D cycle. In a preliminary experiment, normally nocturnal mice were fed during the light phase exclusively (daytime restricted feeding, DRF), over one week. Remarkably, we observed a dramatic reduction in the amplitude of circadian clock gene oscillations in the pituitary gland (data not shown). By contrast, the profiles in the liver were completely phase-inverted as previously reported [15], [16], [17], which denoted that the liver clock was preferentially entrained by feeding-associated cues rather than light-related cues, whereas the response of the pituitary clock appeared more complex.

**Figure 3:**
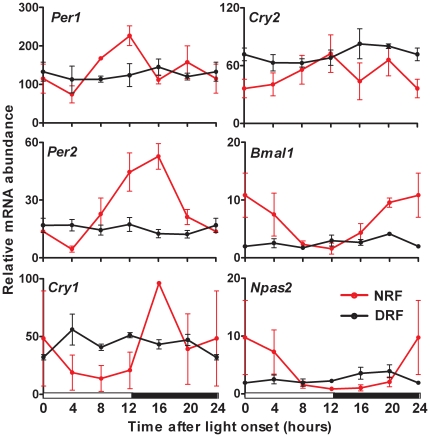
Daytime restricted feeding suppressed the overt rhythmicity of the circadian clockwork in pituitary gland. Circadian clock gene expression profile in the pituitary of mice raised during three consecutive weeks to control nighttime restricted feeding (NRF, red lines) or inverted daytime restricted feeding (DRF, black lines). Values obtained at lights on have been plotted twice (t = 0 and t = 24) for better visualization. The white and black bars below graphs indicate the duration of the light and dark phases, respectively.

In order to ascertain that the pituitary clock eventually reached a steady-state over the course of the restricted feeding protocol, we repeated this experiment and submitted mice to either DRF or nighttime restricted feeding (NRF, control) during three consecutive weeks. Not surprisingly, a complete inversion of clock gene expression profiles was noted between the liver of mice subjected to DRF and NRF (data not shown), as depicted by an average phase shift of 12.52±0.34 hrs (range 11.72 hrs–13.11 hrs, cosinor analysis). Strikingly, after three weeks of DRF, the gene patterns were altered but still not reversed in the pituitary gland. Instead, all transcripts accumulated under DRF at average levels similar to those measured under the NRF protocol, but exhibited a marked loss of overt rhythmicity (Figure 3). Hence, although the alteration of the feeding schedule impacted the expression profile of circadian clock genes in the pituitary, full phase-entrainment was not observed in the gland. Other time cues appeared to be conflicting with meal time in the entrainment of the pituitary gland oscillators.

Because of the heterogeneous composition of the pituitary gland, which contains not less than five different endocrine cell types, the damped rhythms observed in the gland of mice subjected to DRF could result either from a composite response of cell oscillators that would be reset or not by meal time, or from an homogeneously weak entrainment of all pituitary clock cells by food. In order to address this issue, we investigated the pituitary response to DRF at the cellular resolution. Under NRF, the PER1 protein followed a clear rhythm of expression throughout the gland (Figure S1A). PER1 was practically undetectable at ZT8, and accumulated to its maximal level during night around ZT20. At this peak of expression, a much brighter staining was assigned to ACTH-producing cells (Figure S1A, arrows). A positive nuclear PER1 signal was also noted in other endocrine cell types (Figure S1A, arrowheads), including cells that contained growth hormone, prolactin, luteinising hormone or thyroid stimulating hormone (data not shown). Under DRF conditions, PER1 accumulated in most cells of the gland at any time of the light-dark cycle (Figure S1B), in both corticotrophs (arrows) and other cell types (arrowheads). Altogether, these results suggest that the loss of overt rhythm takes place in a large majority of pituitary cells.

### Adrenal glucocorticoids do not inhibit pituitary phase adjustment to feeding schedule

**Figure 4:**
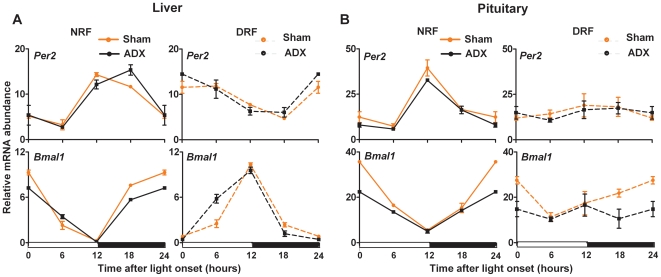
The steady-state response of peripheral circadian oscillators to restricted feeding is resilient to adrenalectomy. Accumulation of clock gene transcripts was measured in sham-operated (red lines) and adrenalectomized (black lines) mice submitted to nighttime restricted feeding (NRF, solid lines) or daytime restricted feeding (DRF, broken lines) during ten days. Values obtained at lights on have been plotted twice (t = 0 and t = 24) for better visualization. The white and black bars below graphs indicate the duration of the light and dark phases, respectively.

A pioneer study reporting the entrainment of peripheral clocks by meal time revealed that circulating glucocorticoids inhibited phase-shifting of the liver clockwork under DRF, as revealed by a faster inversion of clock gene expression patterns in the liver of adrenalectomized mice as compared to sham-operated animals [22]. In that respect, we conjectured that glucocorticoids might potentially prevent the pituitary oscillators from fully adjusting to the feeding schedule. To test this hypothesis we conducted a restricted feeding experiment with mice after ablation of the adrenal gland as the main source of glucocorticoids. The accumulation of Per2 and Bmal1 transcripts, as strongly cycling representatives of the circadian clockwork, was thus assessed in the liver and pituitary gland of sham-operated and adrenalectomized mice sacrificed every 6 hours after 10 days of DRF or NRF (Figure 4). Since DRF-induced phase reversal is completed after two days in liver and kidney of adrenalectomized mice, the duration of our experimental protocol should provide a sufficient delay to conclude whether the rhythm of pituitary clock genes can adjust to the meal schedule in absence of adrenal glucocorticoids.

In accordance with previous report [22], the dramatic decrease of the circulating corticosterone concentration (215.30±24.29 ng/ml vs. 53.76±6.48 ng/ml for the control and adrenalectomized group, respectively, p<0.01 at each time point, two-way ANOVA) did not alter clock gene transcription profiles in the liver of mice raised for ten days under either DRF or NRF schedules (Figure 4A). More interestingly, circadian clock gene patterns induced by restricted feeding in the pituitary were resilient to adrenalectomy (Figure 4B). The daily expression patterns of Per2 and Bmal1 in the pituitary gland of adrenalectomized mice were quite similar to those observed in sham-operated animals. Both transcripts displayed low-amplitude accumulation profiles under DRF that did not correspond to phase-reversal as compared to NRF conditions. Thus, circulating adrenal glucocorticoids did not prevent the pituitary clockwork from adjusting to meal schedule, and the loss of overall cyclic expression of circadian clock genes under DRF unveils that the gland molecular oscillator is regulated through means different from those involved in liver and kidney [22].

## Discussion

Altogether, our results reveal substantial differences in the regulation of circadian clock gene expression between the liver and the pituitary gland. Earlier studies already reported that the photoperiod [32] and the feeding-fasting rhythm [15], [16], [17] differentially regulate peripheral clocks and the central SCN pacemaker. Our data thus unmask a new level of complexity within the circadian system, characterized by the original behavior of the pituitary gland. While most peripheral clocks track the meal time, and the SCN are phase-locked to the light-dark cycle, we here show that the pituitary clockwork integrates signals associated to both time cues. This observation may suggest that the circadian oscillators hosted in the pituitary gland could be functionally involved in between central and peripheral clocks.

The use of cultured explants from transgenic rats expressing the Per1-luciferase reporter as a readout of the circadian clockwork suggested that lungs are not entrained by daytime restricted feeding, but instead remain phase-locked to the light cycle, similarly to the SCN [17]. This observation implies that two different classes of peripheral clocks may exist: one set (liver, kidney, heart, pancreas…) entrained by the feeding-fasting rhythm [15], [16], [17], and a second set (including lung) tracking the light-dark schedule like the SCN [17]. In the pituitary, we found that the expression of one clock protein becomes arrhythmic throughout the gland of mice fed under DRF. This suggests that most pituitary cells track both light onset and meal time, and thus constitute an intermediate third class of circadian clocks However, we can not exclude that “liver-type” and “lung-type” clock cells also coexist within the gland. Other experiments with Per1-luciferase rats already uncovered the complex behavior of the pituitary circadian clockwork [29]. Noteworthy, when transgenic rats were raised under constant light or constant darkness conditions, the phase of the Per1-luciferase oscillations in the pituitary gland differed with respect to preparation time (ZT11 or ZT23), whereas the SCN, the pineal gland and the cornea exhibited more consistent responses [29]. This unique and puzzling response suggested that the pituitary clockwork might be reset by a large variety of signals, including potentially uncontrolled entraining agents in the culture medium.

In mammals, parabiosis experiments have elegantly established that the SCN regulate circadian oscillations in different peripheral organs via distinct pathways. Heart, spleen, and adrenal gland perceive SCN influence exclusively through neural messages, whereas other routes are involved in the entrainment of liver and kidney circadian oscillators [33], [34]. The connections of the SCN with the pre-sympathetic and pre-parasympathetic systems in the hypothalamus provide insight into how the central pacemaker can control several peripheral organs through the autonomous nervous system [19], [20]. The SCN also use blood-borne signals, such as glucocorticoids, to enforce their rhythmicity to the rest of the organism [21], [22], [35]. We found that the removal of adrenal glucocorticoids does not alter the response of the pituitary circadian clockwork to daytime restricted feeding. This observation suggests that an oscillator in the gland is locked to the SCN phase independently of the presence of glucocorticoids. Since the mammalian anterior pituitary is not innervated, other humoral factors must come into play and enable synchronization of the gland oscillators by the SCN [18]. One may suspect that hypothalamic neuroendocrine releasing factors that tune pituitary hormones secretion could be good candidates.

A flexible relative phasing between different circadian oscillators must be a major corollary to the differential entrainment of multiple peripheral clocks by several time cues. The apparent synchronization of most peripheral oscillators, and their average delay of four to six hours as compared to the molecular rhythm in the SCN [15], [17], [18], thus appears as a particular observation under laboratory 12L:12D cycle. Accordingly, we show that the phase lag between circadian mouse oscillators of different tissues and their cycle amplitude are altered by changing photoperiod duration, as previously reported in seasonal hamster [32]. Furthermore, not only the phase of circadian clock gene expression shifts according to photoperiod, but unexpected differences occur in the phase adjustment of various liver clock-controlled genes [36]. Other experiments also suggest that kinetics of resynchronization after jet-lag are different between peripheral tissues, and also between the various clock genes within each organ [35]. Altogether, this complexity of circadian entrainment, even within a single organ, may hamper the possibility of defining a unique internal body time based on genome-wide expression profiles in one peripheral tissue, as this has been proposed [37], [38]. Although this kind of approach has proven extremely powerful under standardized 12L:12D laboratory settings, in which zeitgebers (lights on and meal time) are tightly connected, its application in the field may become challenging.

To some extent, the dual entrainment by light and food is reminiscent of the theoretical model in which two mutually coupled oscillators, tracking dusk and dawn, respectively, are supposed to encode photoperiod [39]. Several studies have suggested that these so-called evening (E) and morning (M) oscillators are hosted in discrete neuronal cell groups in the brain of drosophila [40], [41], and perhaps within the mammalian SCN as well [42], [43], [44]. It is now tempting to speculate that E and M oscillators could be associated in someway to the circadian oscillators entrained by meal time and lights on, respectively, as we defined above. This assumption should make sense at least for nocturnal species that start eating around lights off. In this case, clocks tracking meal time should be also connected to the E oscillator. In accordance with this hypothesis, the cyclic accumulation of Per1 or Dbp transcripts under short or long photoperiod is locked to lights on in lung, and to lights off in heart [32]. Our results show that clock gene expression in mouse liver is also locked to lights off. Since the circadian oscillator in heart and liver, but not in lung, is entrained by the feeding schedule [15], [16], [17], we propose that peripheral organs contain the fingerprint of E and M oscillators. With respect to our results, these two components could be confounded in the pituitary gland.

Thus, the pituitary gland emerges as a compendium of the complexity of peripheral circadian oscillators. The circadian clockwork, expressed in most pituitary cells, may pace a wide number of functions through the various hormones released by the gland. For example, one may speculate that the most appreciable rhythm in PER1 expression in corticotrophs, as compared to other pituitary cell types, might contribute to the circadian activity of the HPA axis. However, the rhythmic clock gene expression in the adrenals does not appear to depend on the pituitary gland [45], but rather on a nervous connection with the SCN [19]. The recent techniques allowing the cell-type specific inactivation of the circadian clockwork [8] will be useful to address the functional relevance of clock genes in the pituitary gland, such as their contribution to the multi-timescale firing activity of endocrine cells [46] and pulsatile hormonal release [47]. Beyond the importance of circadian clock genes in the pituitary, the role of the gland itself within the circadian system still is puzzling. Our results demonstrate that various signals that reset circadian clocks are integrated at the pituitary level. Although pituitary hormones are not crucial for generating rhythms in peripheral tissues [45], they likely contribute to coordinating the system when time cues conflict, such as under time-restricted food availability [24]. The pituitary gland and its circadian oscillators would thus bear a highly adaptive value, at the crossroad of the circadian system.

## Materials and Methods

All animal studies complied with the animal welfare guidelines of the European Community. They were approved by the Direction of Veterinary Services of Hérault, France (Authorizations #34-383 and C34-172-13).

### Animals

Adult male C57/Black6 mice were purchased from Charles River (L'Arbresle, France) and raised under the indicated photoperiod for at least two weeks. Food was given ad libitum, except during restricted feeding protocols, as specified. Mice had free access to tap water.

Before adrenalectomy, analgesia was obtained by an intra-muscular injection of ketoprofen (5 mg/kg). Surgery was performed via a dorso-lumbar approach under isoflurane anaesthesia. The adrenal glands were identified, removed or left in situ (sham), and the incision was closed. Operated animals were allowed to recover during 2 weeks before the restricted feeding protocol, with free access to a 0.9% NaCl solution to preserve their osmotic balance in absence of a secreting source of mineralocorticoids.

### Quantitative Real-time PCR

On the day of experiment, the animals were sacrificed every 4 hours by cervical dislocation. Sham-operated and adrenalectomized mice were sacrificed every 6 hours, and their trunk blood collected for subsequent corticosterone assay [48]. Pituitary gland and liver were rapidly dissected, frozen in liquid nitrogen, and stored at −80°C until use. Total ARN extraction and processing for quantitative real-time PCR were as described previously [47]. The relative expression levels of circadian clock genes were normalized to the Gapdh mRNA accumulation. The sequences of primers used were as follows (forward and reverse, respectively, from 5′ to 3′): Per1, GAAAGAAACCTCTGGCTGTTCCT and GGAATGTTGCAGCTCTCCAAA; Per2, ATCAACCCGTGGAGCAGGAA and GGGAGCTGCGAACACATCCT; Cry1, GTTCGCCGGCTCTTCCA and ATCCTCAAGACACTGAAGCAAAAA; Cry2, GGGACTCTGTCTATTGGCATCTG and GTCACTCTAGCCCGCTTGGT; Bmal1, GCAGTGCCACTGACTACCAAGA and TCCTGGACATTGCATTGCAT; Clock, CACAGCGGAGGTCGTCCTT and GACATCGCTGGCTGTGTTAATG; Npas2, CACTCGGAAAATGGACAAAACC and TGAGACTTCATTGTGTTTCTGCAA; Gapdh, GGAGCGAGACCCCACTAACA and ACATACTCAGCACCGGCCTC.

### Immunohistofluorescence

Mice received an overdose of pentobarbital and were perfused transcardially with 4% paraformaldehyde. Pituitary glands were dissected, and 50-µm thick coronal sections were prepared with a vibratome. Free-floating pituitary sections were incubated with anti-PER1 primary serum (1/2000, donated by David Weaver, rabbit #1177 [49]) and an antibody raised in guinea-pig against either growth hormone, prolactin, luteinising hormone or thyroid stimulating hormone (1/8000, from Alfred Parlow). The primary antibodies were detected with Cy3-conjugated anti-rabbit and Alexa 488-conjugated anti-guinea pig secondary antibodies (1/2000, Molecular Probes). The fluorescent staining was visualized and imaged with an ApoTome microscope (Zeiss).

### Data analysis

Normalized values of circadian clock gene expression levels where processed for analysis with the Igor Pro 5 software (Wavemetrics). The circadian phase and mean level of each gene profile were then estimated by cosinor analysis, using the following equation in the curve fitting: f(t) = M+A. cos (2π. (t - ϕ)/24), (where t =  time in hours, M =  mesor, A =  amplitude, ϕ =  peak time).

Statistical analysis was performed with the Prism 5 software (GraphPad). The liver and pituitary circadian oscillators were compared by Wilcoxon matched-pairs signed rank test, where the in liver and pituitary expression profiles of each gene were matched pairs. Circulating corticosterone levels in sham-operated and adrenalectomized mice were compared with two-way ANOVA, where the operation protocol and the time of sample collection were the variables. All values in the text are expressed as mean ± standard deviation. In the figures, transcript accumulation data are plotted as mean ± standard error of the mean.

## Supporting Information

Figure S1 **Expression of the circadian clock protein PER1 in the mouse pituitary gland.** Accumulation of PER1 protein was assessed by immunofluorescence in pituitary sections from mice submitted to nighttime feeding (NRF) or daytime feeding (DRF), sacrificed eight (ZT8, left column) or twenty (ZT20, right column) hours after light onset. (**A**) Under NRF, PER1 (red) is barely detectable at ZT8, and expressed throughout the gland at ZT20. PER1 is expressed in ACTH-containing cells (arrows, green) and other cell types (arrowheads). (**B**) Under DRF, expression levels of PER1 were similar throughout the gland at both time points. Insets show magnified details from merged images.
(TIF)
