# Supplementary figures and images for: The Comparison between Circadian Oscillators in Mouse Liver and Pituitary Gland Reveals Different Integration of Feeding and Light Schedules
Source: PLoS One. 2010 Dec 15;5(12):e15316. doi: 10.1371/journal.pone.0015316 (PMC3002272; doi:10.1371/journal.pone.0015316)

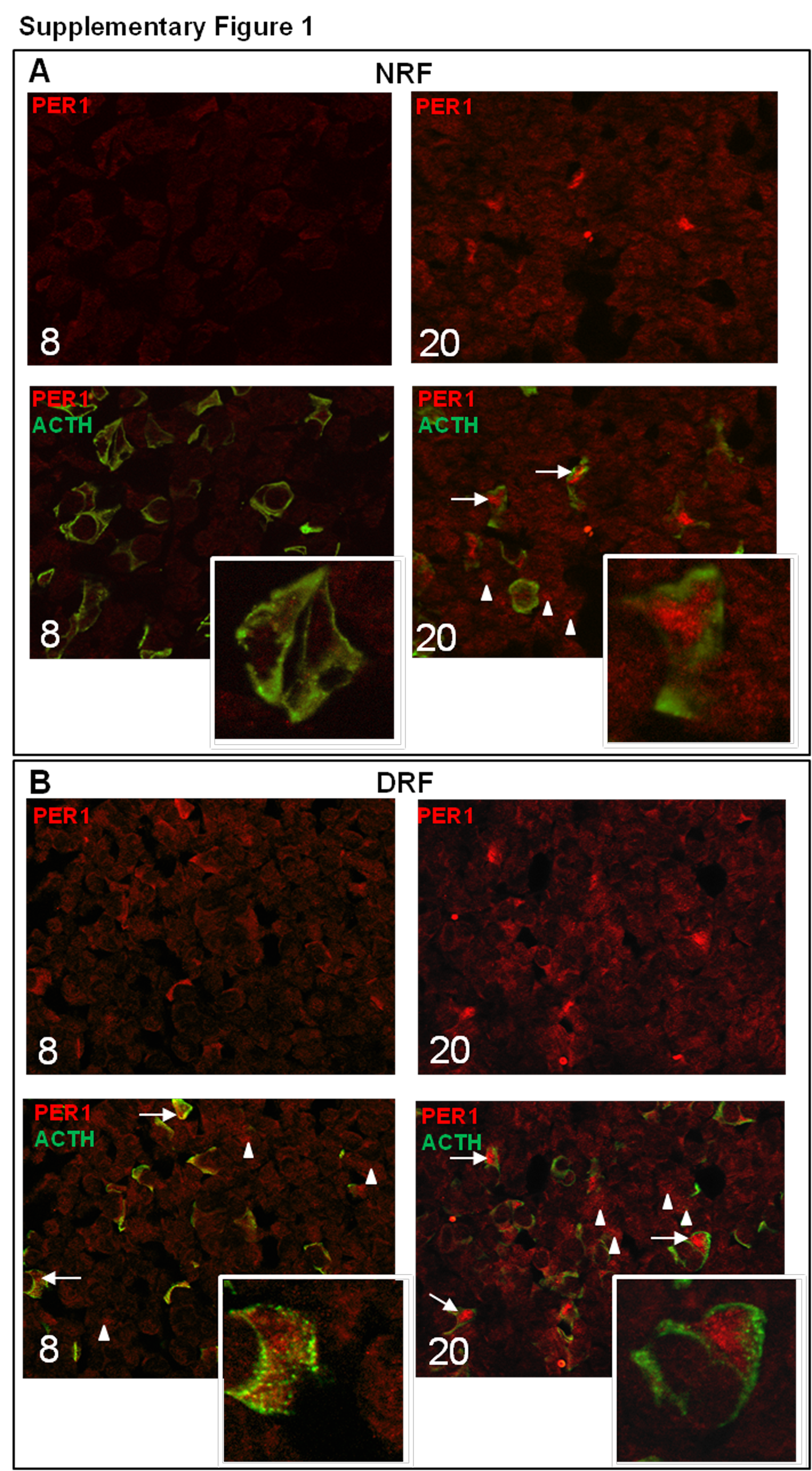

Supplement: Figure S1 — Expression of the circadian clock protein PER1 in the mouse pituitary gland. Accumulation of PER1 protein was assessed by immunofluorescence in pituitary sections from mice submitted to nighttime feeding (NRF) or daytime feeding (DRF), sacrificed eight (ZT8, left column) or twenty (ZT20, right column) hours after light onset. (A) Under NRF, PER1 (red) is barely detectable at ZT8, and expressed throughout the gland at ZT20. PER1 is expressed in ACTH-containing cells (arrows, green) and other cell types (arrowheads). (B) Under DRF, expression levels of PER1 were similar throughout the gland at both time points. Insets show magnified details from merged images. (TIF) [file pone.0015316.s001.tif]
